# Large-scale environmental DNA survey reveals niche axes of a regional coastal fish community

**DOI:** 10.1038/s41598-025-31307-4

**Published:** 2026-02-16

**Authors:** Yutaka Osada, Masaki Miya, Hitoshi Araki, Hideyuki Doi, Akihide Kasai, Reiji Masuda, Toshifumi Minamoto, Satoquo Seino, Teruhiko Takahara, Satoshi Yamamoto, Hiroki Yamanaka, Mitsuhiro Aizu-Hirano, Keiichi Fukaya, Takehiko Fukuchi, Ryo O. Gotoh, Masakazu Hori, Midori Iida, Tomohito Imaizumi, Tadashi Kajita, Takashi Kanbe, Tanaka Kenta, Yumi Kobayashi, Tomohiko Matsuura, Hiroki Mizumoto, Hiroyuki Motomura, Hiroaki Murakami, Kenji Nohara, Shin-ichiro Oka, Tetsuya Sado, Hiroshi Senou, Koichi Shibukawa, Tomoki Sunobe, Hiroshi Takahashi, Koji Takayama, Katsuhiko Tanaka, Hisashi Yamakawa, Satoru Yokoyama, Seokjin Yoon, Michio Kondoh

**Affiliations:** 1 Advanced Institute for Marine Ecosystem Change, Tohoku University, Sendai, Miyagi 980-8578 Japan; 2 Natural History Museum and Institute, Chiba, Chiba 260-8681 Japan; 3 Research Faculty of Agriculture, Hokkaido University, Sapporo, Hokkaido 060-8589 Japan; 4 Graduate School of Informatics, Kyoto University, Yoshida-honmachi, Sakyo-ku, Kyoto, 606-8501 Japan; 5 Faculty of Fisheries Sciences, Hokkaido University, Hakodate, 041-8611 Hokkaido Japan; 6 Maizuru Fisheries Research Station, Field Science Education and Research Center, Kyoto University, Maizuru, Kyoto 625-0086 Japan; 7 Graduate School of Human Development and Environment, Kobe University, Kobe, Hyogo 657-8501 Japan; 8 Graduate School of Engineering, Kyushu University, Fukuoka, Fukuoka 819-0395 Japan; 9 Faculty of Life and Environmental Sciences, Shimane University, Matsue, Shimane 690-8504 Japan; 10 Estuary Research Center, Shimane University, Matsue, Shimane 690-8504 Japan; 11 Institute for Agro-Environmental Sciences, NARO, Tsukuba, Ibaraki 305-8604 Japan; 12 Faculty of Science and Technology, Ryukoku University, Otsu, Shiga 520-2194 Japan; 13 Research Center for Biodiversity Science, Ryukoku University, Otsu, Shiga 520-2194 Japan; 14 The Institute of Statistical Mathematics, Tachikawa, Tokyo, 190-8562 Japan; 15 National Institute for Environmental Studies, Tsukuba, Ibaraki 305-8506 Japan; 16 Fisheries Stock Assessment Center, Japan Fisheries Research and Education Agency, Yokohama, Kanagawa 236-8648 Japan; 17 Marine Biological Station, Sado Island Center for Ecological Sustainability, Niigata University, Sado, Niigata 952-2135 Japan; 18 Usujiri Fisheries Station, Field Science Center for Northern Biosphere, Hokkaido University, Hakodate, Hokkaido 041-1613 Japan; 19 National Research Institute of Fisheries Engineering, Kamisu, Ibaraki 314-0408 Japan; 20 Iriomote Station, Tropical Biosphere Research Center, University of the Ryukyus, Yaeyama, Okinawa 907-1541 Japan; 21 Sugadaira Montane Research Station, Mountain Science Center, University of Tsukuba, Ueda, Nagano 386-2204 Japan; 22 Salmon Research Department, Fisheries Resources Institute, Japan Fisheries Research and Education Agency, Sapporo, Hokkaido 062-0922 Japan; 23 The Kagoshima University Museum, Korimoto, Kagoshima, 890-0065 Japan; 24 Graduate School of Agricultural Science, Tohoku University, Sendai, Miyagi 980-8572 Japan; 25 School of Marine Science and Technology, Tokai University, Shimizu, Shizuoka 424-8610 Japan; 26 Okinawa Churashima Foundation, Motobu, Okinawa 905-0206 Japan; 27 Kanagawa Prefectural Museum of Natural History, Odawara, Kanagawa 250-0031 Japan; 28 Museum of Natural and Environmental History, Shizuoka, Shizuoka 422-8017 Japan; 29 Tateyama Station, Field Science Center, Tokyo University of Marine Science and Technology, Tateyama, Chiba 294-0308 Japan; 30 National Fisheries University, Shimonoseki, Yamaguchi 759-6595 Japan; 31 Graduate School of Science, Tokyo Metropolitan University, Hachioji, Tokyo, 192-0397 Japan; 32 Kazusa DNA Research Institute, Kisarazu, Chiba 292-0818 Japan; 33 National Institute of Fisheries Science, Busan, 46083 Republic of Korea; 34 Graduate School of Life Sciences, Tohoku University, Sendai, Miyagi 980-8578 Japan; 35 NIES Lake Biwa Branch Office, National Institute for Environmental Studies, Otsu, Shiga, 520-0022 Japan

**Keywords:** Biodiversity, Biogeography, Community ecology, Ecological modelling

## Abstract

The concept of the ecological niche, defined as the basic habitat requirements for a species, is central to understanding species geographic distributions and predicting their responses to environmental change. However, identifying the essential niche for large regional communities remains a challenge because niche axes can be “hidden” by the complexity of the underlying ecological processes. Here, applying advanced species distribution modelling to nationwide environmental DNA survey data, we identified hidden niche axes of the Japanese coastal fish community and investigated the response diversity to these axes. Our survey detected 1,220 coastal fish species. The hidden niche axes collectively explained most of the variation in fish biodiversity and revealed five biogeographic boundaries for the regional community. These niches of the Japanese fish community may primarily relate to several processes due to ocean currents, such as environmental filters, transport from source areas and dispersal barriers. We also found that the response diversity to niche axes was positively correlated with species richness, although local communities with particularly high response diversity were geographically biased. A better understanding of the niche axes of the regional ecological community should help to mitigate the loss of biodiversity and ecosystem services caused by ongoing environmental change.

**Supplementary Information:**

The online version contains supplementary material available at 10.1038/s41598-025-31307-4.

## Introduction

The ecological niche is a fundamental concept for explaining the geographic distributions and coexistence of species^1–6^. According to a widely accepted definition^3^, the niche of a species is represented as a multidimensional space with abiotic and biotic axes in which populations can persist. Numerous field and experimental studies have underscored the critical role of the ecological niche in shaping biodiversity from both ecological and evolutionary perspectives^6–10^. In most studies, the axes of niche space can be explored by modelling species distributions using abiotic and biotic variables^6,11^. However, identifying the essential niche axes for a large regional community with diverse species remains challenging, mainly because niche axes can be “hidden” when the underlying ecological processes are too complex to measure (e.g. local species interactions, dispersal limitation, and evolutionary history). The existence of hidden niche axes obscures our understanding of how regional communities will respond to environmental change. Given the current biodiversity crisis due to habitat degradation and global warming^12,13^, the identification of hidden niche axes is an urgent issue. The aim of this study is to identify the hidden niche axes of the nationwide coastal fish community by combining a large-scale environmental DNA (eDNA) biodiversity survey with a recently developed joint species distribution model^14–16^.

**Fig. 1:**
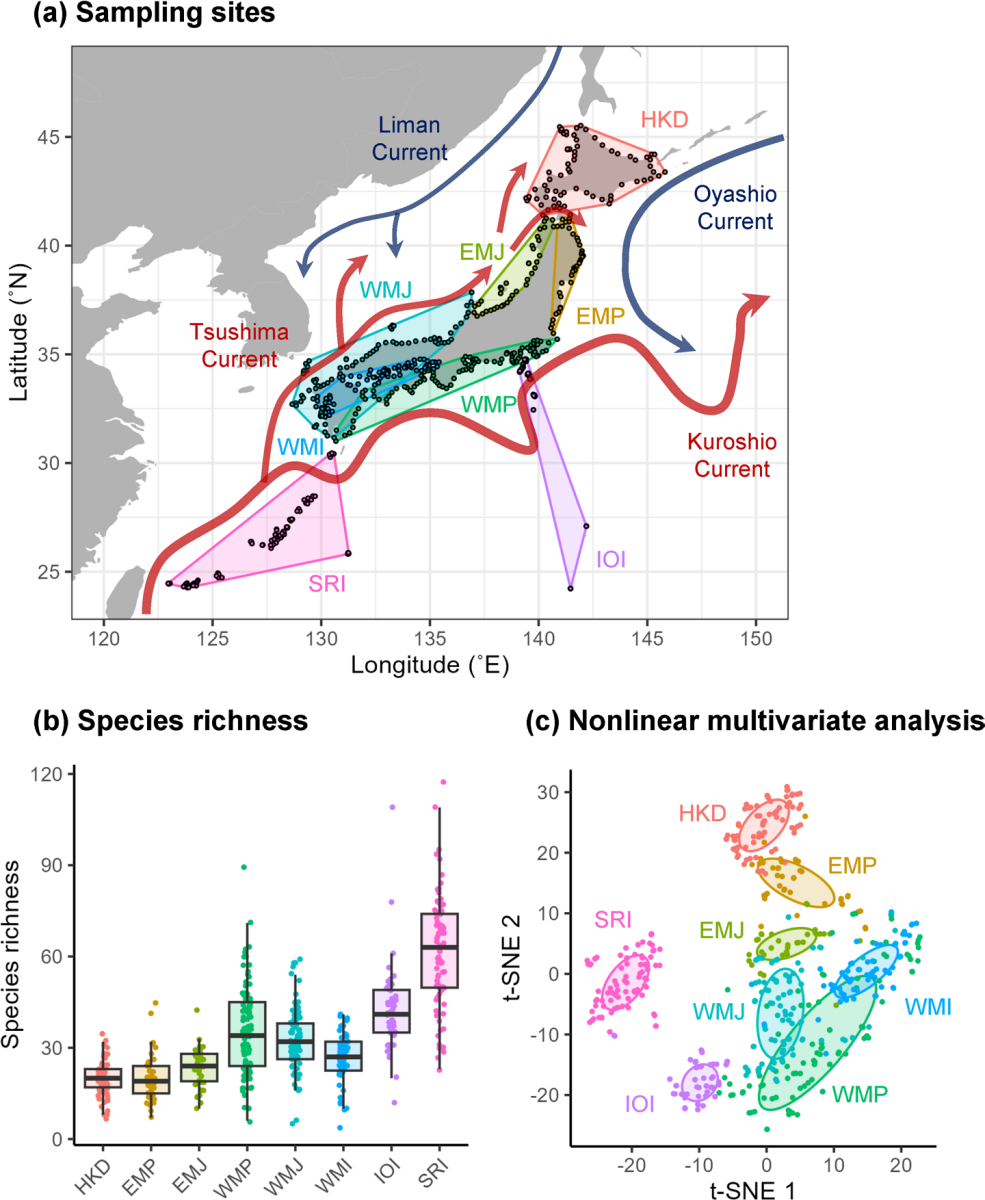
(a) Sampling sites (points) were geographically grouped into eight districts: Hokkaido Islands (HKD, *n* = 71), East Main Islands along the Pacific Ocean (EMP, *n* = 39), East Main Islands along the Japan Sea (EMJ, *n* = 39), West Main Islands along the Pacific Ocean (WMP, *n* = 95), West Main Islands along the Japan Sea (WMJ, *n* = 90), West Main Islands along inland sea (WMI, *n* = 63), Izu-Ogasawara Islands (IOI, *n* = 51), and Satsuma-Ryukyu Islands (SRI, *n* = 80). Kuroshio and Tsushima warm currents (red arrows) and Oyashio and Liman cold currents (blue arrows) result in high productivity in the study area. (b) Comparisons of rarefied species richness and (c) nonlinear multivariate analysis (t-SNE) were conducted. Coloured points represent sampling sites from different districts; 50% probability ellipses are shown to clarify the spatial structure of the fish communities in the multivariate analysis.

eDNA metabarcoding techniques have improved the efficiency and comprehensiveness of biodiversity surveys, especially for fish, based on analyses of extra-organismal DNA suspended in the surrounding environment^17–19^. Their technical developments provide an opportunity to explore the hidden niche axes of regional communities, as eDNA surveys can cover extensive areas in a relatively short period. Here, we conducted a nationwide eDNA survey to investigate the fish community in the coastal area of the Japanese archipelago. This region is well-known for its high fish diversity, not only because the wide range of climatic zones provides a variety of coastal environments, but also because four major ocean currents result in high productivity in this area^20,21^. Our eDNA biodiversity survey was conducted at 528 sampling sites along the Japanese coastline from June to August 2017 (Fig. 1 and Supplementary Table S1). Of these sites, 318 were located on the four major islands (Hokkaido, Honshu, Shikoku, and Kyushu) and the remaining 210 were located on 60 smaller, remote islands. The study area extended over 19,000 km of coastline, encompassing a climate range from subtropical to temperate and subarctic zones (24.2°N–45.5°N).

In this study, we adopted the practical definition of ecological niche axes for regional communities: it contributes to the population persistence of the majority of compositional species. In this definition, ecological niche can be related to various ecological processes, including not only abiotic factors (environmental filter)^1,3^ but also biotic factors (species interactions, dispersal limitation, evolutionary history, etc.)^2,4,5^. It is noteworthy that because niche axes were explored by modelling species distributions, it may be difficult to identify niche axes whose contributions to the population persistence fluctuate over time and space^6^.

## Results and discussion

After completing DNA extraction, parallel sequencing, and taxonomic assignment (see Methods and Supplementary Information S1), we detected 1,220 MOTUs (molecular operational taxonomic units; hereafter species) of coastal fishes from our survey, spanning 148 families and 501 genera (Supplementary Tables S2–S4). Local species richness at each site varied from 6 to 118 with a median of 34 species. The well-known latitudinal biodiversity gradient^22,23^ was observed, with lower richness in the north and higher richness in the south (Fig. 1). Specifically, Hokkaido Islands (HKD) and East Main Islands along the Pacific Ocean (EMP) had the lowest species richness, while West Main Islands along the Pacific Ocean (WMP), Izu-Ogasawara Islands (IOI) and Satsuma-Ryukyu Islands (SRI) had the highest species richness. A nonlinear multivariate analysis (t-SNE) of the entire fish community revealed clear geographically associated clusters (Fig. 1). The subtropical communities in IOI and SRI and the subarctic communities in HKD and EMP formed completely separate clusters, and these clusters were connected by the temperate communities in other regions. The coastal fish communities included many uncommon species that occupied fewer than six sites but accounted for more than half of the total species richness (670 out of 1,220 species).

**Fig. 2:**
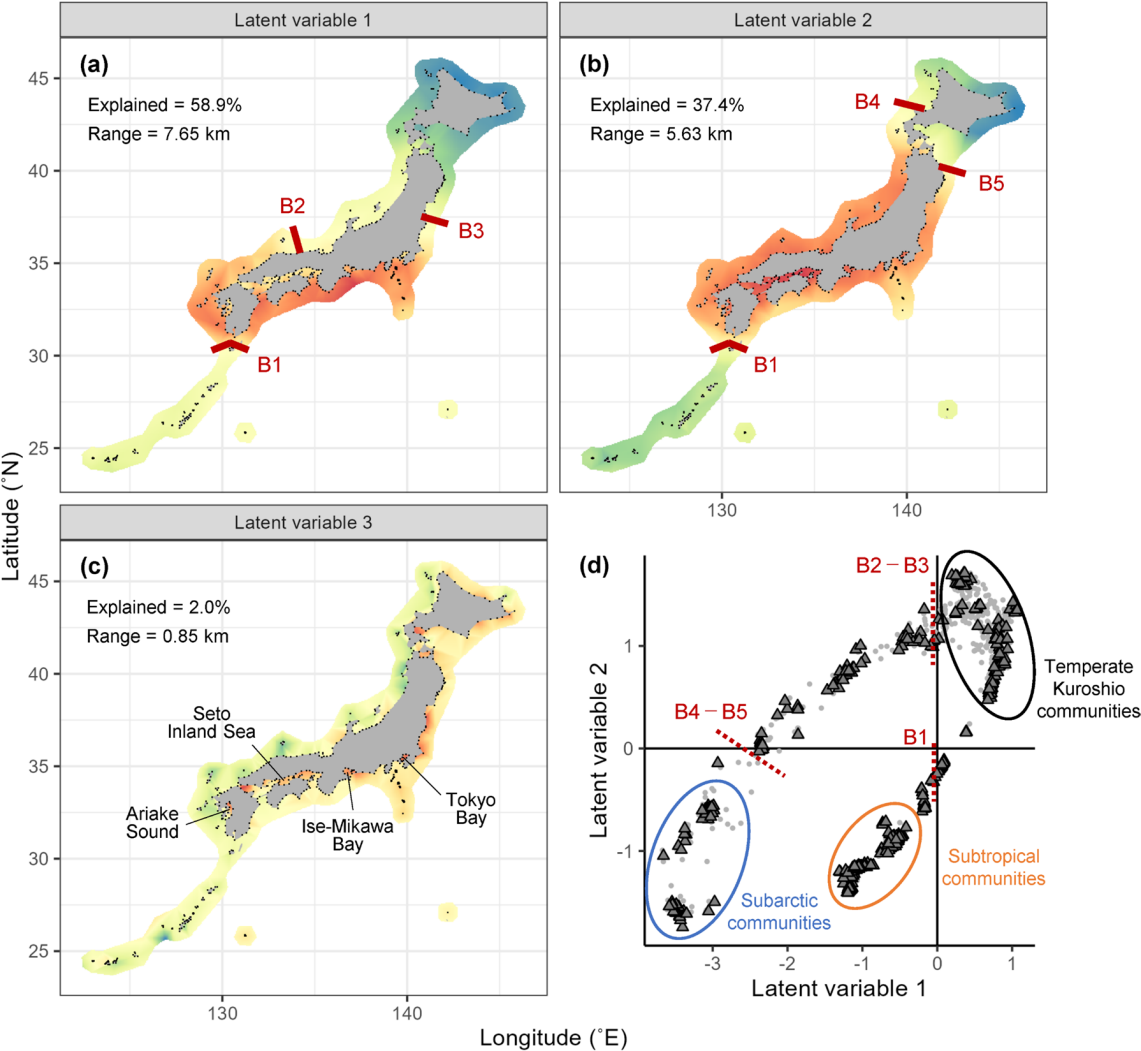
(**a–c**) Geological variation in latent variables estimated as hidden niche axes. Colour gradient from blue to red represents the values of scaled latent variables. Areas with similar colours exhibit similar niche values. Points represent sampling sites. For latent variables 1 and 2, we identified five hypothetical boundaries between distinct local communities (B1–B5; red lines). The latent variables explained 58.9%, 37.4%, and 2.0% of the total variance, respectively. Range represents the scale of spatial autocorrelation obtained from Matérn covariance. (**d**) Niche space with latent variables 1 and 2 as axes. Gray points represent the values for the sampling sites and triangles represent the niche centres of the analysed species. Red dotted lines represent zero-value lines corresponding to above five hypothetical boundaries. Ellipses represent representative species types based on the niche space.

To infer hidden niche axes that shape the regional coastal fish community, we employed a generalised linear latent variable model (GLLVM)^14–16^. This model estimates species responses not only to environmental covariates (e.g. seawater temperature and salinity) but also to spatially referenced latent variables for explaining species distribution ranges (see Methods). Spatially referenced latent variables, estimated from shared residuals of species co-occurrence patterns^24,25^, can account for the hidden axes of shared niche space (e.g. unmeasured environments, local species interactions, dispersal limitation and evolutionary history). To avoid the identification of false niche axes due to model overfitting, we fitted the model with 80% of the sampling sites and validated the fitted model with the remaining 20% to compare the predictive performance of 18 candidate models (see Methods). The best-fit model had three latent variables, which collectively explained 98.3% of the variance in species co-occurrence (58.9%, 37.4% and 2.0% for each latent variable; Fig. 2). Measured environmental covariates explained only 1.7% of the variance (1.0% for temperature and 0.7% for salinity).

The first and second latent variables, most influential on the regional fish community of the Japanese coast, revealed five hypothetical biogeographic boundaries between distinct local communities (Fig. 2, B1–B5; see Methods for boundary identification). These hypothetical boundaries aligned with those documented in previous studies^26–28^. Boundary B1 is known as the Osumi Line, a dispersal barrier due to the broad and fast-flowing Kuroshio Current, by which many sister species are separately distributed only off the Japanese mainland or the Satsuma-Ryukyu Islands^26^. Boundaries B2–B3 and B4–B5 correspond to the boundaries of three marine provinces (Sino-Japanese, Oriental, and Kuril Provinces)^27,28^. The fish fauna of the area south of Boundary B2–B3 is likely explained by the transport of tropical fishes from the northern Philippines and eastern Taiwan by warm currents, while that of the area north of Boundary B4–B5 is explained by the transport of subarctic fishes from the Russian Far East by cold currents. Boundary B2 appeared to arise from the change in coastal depth. The shallow southern area is dominated by warm water from Tsushima Current^29^ and the deep northern area is dominated by cold water from the abyssal circulation of the Japan Sea^30^. Interestingly, these boundaries may also explain the distributions of large seagrasses and seaweeds^31^ and terrestrial plants^32,33^. The ecological processes underlying these hidden niche axes of coastal fishes may be important for other taxonomic groups with diverse life histories.

Using the first and second latent variables as axes, we reconstructed the niche space in which the species niche centres were located (Fig. 2d). We observed that for the Japanese coastal fish community, the niche centres of many species were in the subtropical (35.3% of all species) and temperate Kuroshio regions (25.4%), whereas fewer species had niche centres in the subarctic region (10.4%). This distribution of niche centres was consistent with the latitudinal variation in species richness (Fig. 1). Thus, our analysis reemphasises the critical role of ecological niches in characterising regional biodiversity^6^. When the niche space was constructed for each family with more than five species (Supplementary Figure S1), we found that for some families, the niche centres occupied only a portion of the niche space. For example, most species of Acanthuridae, Chaetodontidae, Balistidae and Monacanthidae, which inhabit mainly tropical seas and coral reefs, occupied the niche space from the subtropical to temperate Kuroshio regions. This pattern suggests that these niche axes play an important role in driving regional spatial turnover at the family level.

The third latent variable revealed a niche axis associated with geographically scattered habitats that are important to the regional fish community (Fig. 2c and Supplementary Figure S2). These habitats included enclosed inland seas, such as Ariake Sound, Seto Inland Sea, Ise-Mikawa Bay, and Tokyo Bay, which have lower salinities and colder temperatures than those of adjacent open seas. These enclosed inland seas harbour distinct species that may have originated from the geographic isolation of the Japan Sea about 6 million years ago^33^. Thus, the niche space of the Japanese coastal fish community appears to be shaped by the interplay of environmental factors as well as dispersal limitation and evolutionary history. This may explain why it is often difficult to capture hidden niche axes with a limited set of measured variables. Of note, we did not find niche axes corresponding to local species interactions (i.e. the Eltonian niche)^2^. This may be explained by the Eltonian noise hypothesis^6^, which predicts that the effect of species interactions will generally take place at finer spatial scales and not necessarily across broad biogeographic regions.

**Fig. 3:**
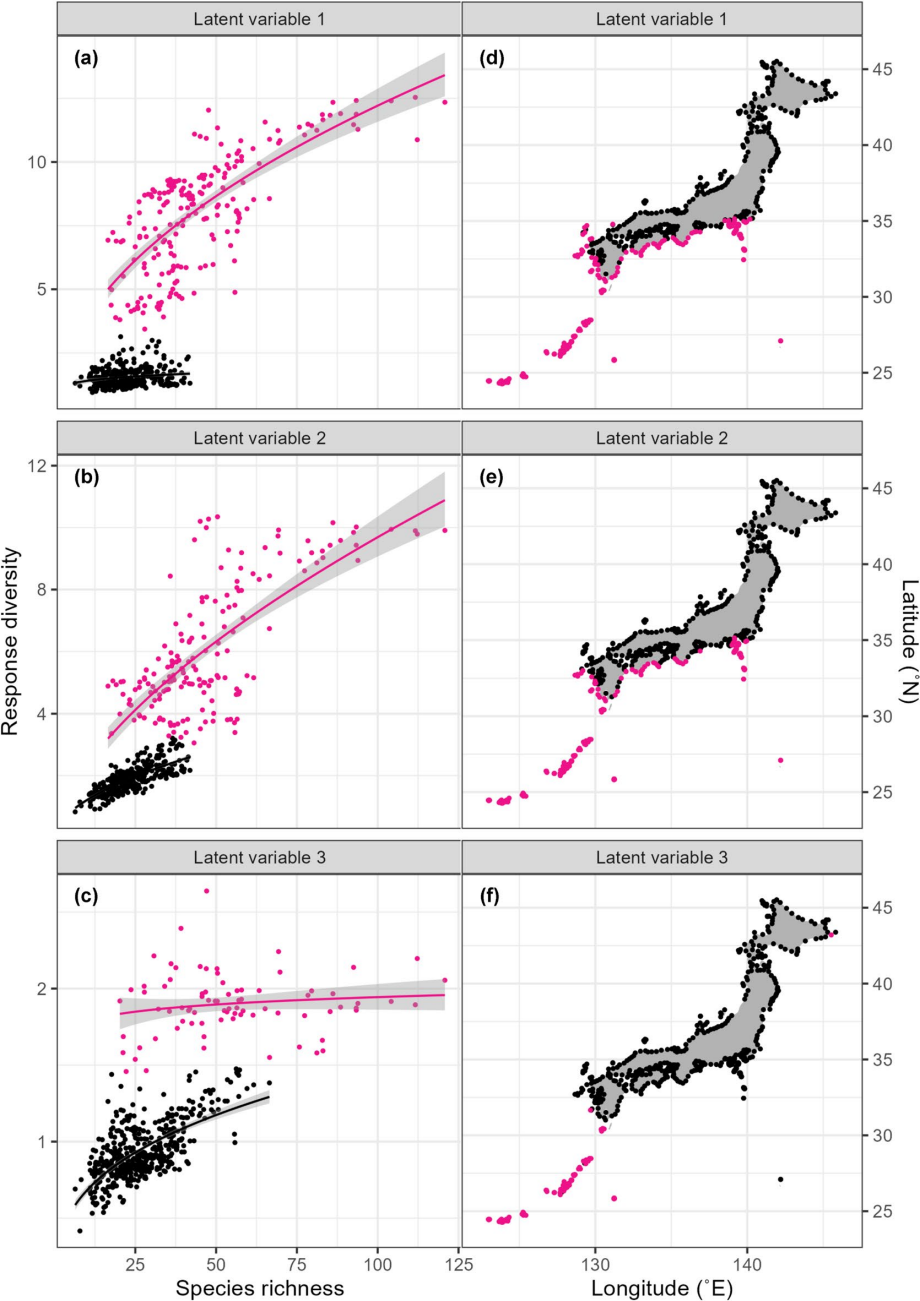
(**a–c**) Relationship between expected species richness and response diversity to each latent variable (i.e. niche axis), calculated from the species-specific coefficients of the generalised linear latent variable model. Local fish communities at sampling sites (points) were classified into groups with high and low response diversity (red and black, respectively) using spectral clustering. Lines and shaded areas represent regression lines and standard deviations. (**d–f**) Locations of sites with high response diversity (red points) and low response diversity (black points).

Finally, we investigated the response diversity of local fish communities to the latent variables (Fig. 3). Response diversity, defined as the variation in responses to environmental change among species in a community, is a key determinant of the stability of ecosystem functions^34,35^. Community-level functions are expected to be less variable in communities with high response diversity. As expected, we found a positive relationship between species richness and response diversity (Fig. 3). However, species richness did not fully explain the variation in response diversity. When local fish communities were classified into groups with high and low response diversity by clustering, the communities with high response diversity were geographically biased, particularly along the Pacific coast of western Japan (Fig. 3 and Supplementary Figure S3). This geographic bias may be explained by habitat diversity provided by complex coastal inlets and coral reefs, in addition to fish migration from lower latitudes due to the Kuroshio current. Further studies are needed to understand the mechanisms underlying the increased response diversity of the coastal fish community in specific geographic regions.

Our model showed that field-measured environmental variables (seawater temperature and salinity) exhibited surprisingly low explanatory power for the regional fish community. To investigate the reason, we conducted additional GLLVM analysis with satellite-measured variables as environmental covariates (see Methods and Supplementary Information S2). Satellite-measured variables include annual minimum and maximum sea surface temperatures (SST), mean chlorophyll a concentration, and mean particulate inorganic carbon concentration. Although the predictive performance of the additional GLLVM was considerably less than original GLLVM, the results showed that environmental covariates explained about 80% of the variation in fish biodiversity, while latent variables explained the remaining 20%. In particular, annual SST and chlorophyll a concentration explained most of this variation (about 50% and 20%, respectively). This suggests that long-term environmental factors may be more critical for the regional fish community than environmental factors at the time of sampling. Thus, incorporating appropriate environmental variables as covariates can reduce the variation explained by latent variables, which require careful interpretation.

Additionally, the importance of identifying niche axes based on measured environmental variables must be recognised. Measured environmental niche axes will provide more robust predictions outside of study areas and under future scenarios than hidden niche axes identified by latent variables. As shown in our study, the point of using latent variables is that they allow us to interpret niche axes that are difficult to measure. Our approach, which uses both measured environmental variables and hidden latent variables as covariates, will be a good starting point for identifying ecological niche of regional communities.

Coastal ecosystems are highly productive and diverse ecosystems^36^. They cover only 11% of oceans but support 90% of the catch from marine fisheries^37^. Our study demonstrates the application of nationwide eDNA survey data for the identification of hidden niche axes shaping regional fish diversity and for understanding underlying ecological processes in a coastal ecosystem. In particular, ocean currents likely relate to the underlying ecological processes as environmental filters, transport from source area, and dispersal barriers. Global warming may therefore have a serious impact on the Japanese coastal ecosystem because it not only directly increases seawater temperatures but also indirectly alters current water transport through the global climate oscillation^38^. To predict changes in the regional fish community due to global warming, future efforts must be made to explicitly incorporate these ecological processes into atmosphere-ocean coupled general circulation models^39^. The United Nations recently declared 2021–2030 the Decade on Ecosystem Restoration, with the aim of preventing, halting and reversing the degradation of ecosystems on every continent and in every ocean^40^. eDNA surveys based on recently developed metabarcoding techniques will undoubtedly contribute to achieving this ambitious goal.

### Methods

#### Sampling site selection

The Japanese archipelago extends over 3,000 km along the northeastern coast of the Eurasian continent, facing the Sea of Okhotsk, Pacific Ocean, Seto Inland Sea, East China Sea, and Japan Sea. To sample the vast coastal areas of Japan, 528 sampling sites were selected (Fig. 1a), covering a wide range of the climatic zones from subtropical to temperate and subarctic (24.227–45.552˚N, 122.955–145.815˚E). For the four main islands (Hokkaido, Honshu, Shikoku, and Kyushu), 318 sites were located along the concatenated coastlines with an average distance of 60 km between each site. Of the more than 6,000 smaller islands around the main islands, 60 islands were selected for more comprehensive geographic coverage. The 210 sites were located on these 60 islands 1–1,000 km offshore from the main islands with an average distance of 27 km between each site. Exact site locations were determined (1) to evenly divide the lengths of the coastlines, (2) to ensure safety during the water sampling and (3) to safeguard against contamination from exogenous DNA. It is noteworthy that fish eDNA is considered to reflect its local abundance in marine environments^41^. Therefore, given that the distance between sampling sites is sufficiently large, the transport of eDNA itself by currents will have little impact on our results.

## Water sampling and on-site filtration

Seawater sampling was conducted at 528 sites during the summer of 2017, from June 5 to August 18 (Supplementary Table S1). At each sampling site, surface seawater was collected by casting a polypropylene bucket tied to a 15-m rope onto the sea surface. Seawater collection was repeated ten times to minimise sampling bias at each site. Disposable gloves were worn on both hands during water sampling, and the bucket inside and the tip of the rope were thoroughly decontaminated with a foam-style 10% bleach solution to reduce contamination risk. On-site filtration was performed by two researchers to obtain duplicate samples. For a single bucket cast, each researcher filtered 50 ml of seawater twice using a Sterivex filter cartridge (pore size 0.45 μm; Merck Millipore, Billerica, MA) and a 50-ml disposable syringe (Terumo Corp., Tokyo, Japan). The final filtration volume reached 1,000 ml × 2 with ten bucket casts. When the filter was clogged before reaching 1,000 ml filtration, the total volume of water filtered was recorded (200–900 ml from 28 sites; Supplementary Table S1). After on-site filtration, an outlet port of the filter cartridge was sealed with Parafilm (LMS, Tokyo, Japan), and 1.6 ml of RNA*later* (Thermo Fisher Scientific, Waltham, MA) was added to the cartridge from an inlet port using a disposable capillary pipette (As One Corp., Osaka, Japan) to prevent eDNA degradation. The inlet port was sealed with either the film or a cap for preservation. A filtration blank (FB) was prepared by filtering 500 ml of purified water in the same manner at the end of each sampling day. The filtered cartridges were transported to the laboratory in a portable cooler and then kept at − 20 °C in the freezer until eDNA extraction.

## DNA extraction

A physically separated workspace was prepared for DNA extraction and subsequent column-based steps. The workspace and equipment were sterilised with hypochlorite solution before DNA extraction. To prevent contamination and the loss of environmental DNA, low-retention filtered pipette tips and microtubes were used. DNA extraction was performed according to the previously developed method^42^ with slight modifications. After removing redundant seawater and RNA*later* from the filter cartridge by an aspirator, crude eDNA was extracted from the filter membrane using proteinase K buffer, consisting of 220 µl of PBS, 20 µl of Proteinase K, and 200 µl of Buffer AL, included in a DNeasy Blood & Tissue Kit (Qiagen, Hilden, Germany). After sealing proteinase K buffer into the filter cartridge and stirring at 56 °C for 10 min, the extracted DNA was collected and further purified using the kit. The final elution volume of Buffer AE from the column was 200 µl. During this process, an extraction blank (EB) was prepared using Milli-Q Water-enclosed filter cartridge.

## Paired-end library Preparation and sequencing

The workspace and equipment in the pre-PCR area were sterilised with hypochlorite solution before library preparation. Low-retention filtered pipette tips and microtubes were used and pre- and post-PCR manipulations were performed in two different dedicated rooms to safeguard against carryover contamination. Two-step PCR (first-round PCR and index PCR) was applied for paired-end next generation sequencer (NGS) library preparation, following previously developed methods^43,44^. For the first-round PCR (1st PCR), a mixture of two primer pairs was used: MiFish-U-forward (5′–ACA CTC TTT CCC TAC ACG ACG CTC TTC CGA TCT NNN NNN GTC GGT AAA ACT CGT GCC AGC–3′), MiFish-U-reverse (5′–GTG ACT GGA GTT CAG ACG TGT GCT CTT CCG ATC TNN NNN NCA TAG TGG GGT ATC TAA TCC CAG TTT G–3′), MiFish-E-forward-v2 (5′–ACA CTC TTT CCC TAC ACG ACG CTC TTC CGA TCT NNN NNN RGT TGG TAA ATC TCG TGC CAG C–3′) and MiFish-E-reverse-v2 (5′–GTG ACT GGA GTT CAG ACG TGT GCT CTT CCG ATC TNN NNN NGC ATA GTG GGG TAT CTA ATC CTA GTT TG–3′). The 1st PCR was carried out with a 12-µl reaction volume in eight replications using a strip of eight 0.2-ml tubes. A 1st PCR blank (1B) was also prepared during this process. To reduce the experiment costs, a single 12-µl reaction was prepared for each type of blank (FB, EB, and 1B). After the 1st PCR, equal volumes of the PCR products were pooled from the eight replications in a single tube and purified using a GeneRead Size Selection Kit (Qiagen, Hilden, Germany). Subsequently, the purified target products (ca. 300 bp) were quantified using TapeStation 2200 (Agilent Technologies, Tokyo, Japan). After diluting samples to 0.1 ng/µl with Milli-Q water, 1.5 µl of each diluted product was subjected to the index PCR (2nd PCR) in a 12-µl reaction volume to append dual-indexed sequences and flow cell-binding sites for NGS platform. For the three types of blanks (FB, EB, and 1B), the 1st PCR products were purified in the same manner but were not quantified. They were diluted at the average dilution ratio of the positive samples to be used as templates for the 2nd PCR. The following two primers were used for the 2nd PCR to append dual-indexed sequences (eight nucleotides indicated by Xs) and flow cell-binding sites for the MiSeq platform (5′–ends of the sequences before eight Xs): 2nd-PCR-forward (5–AAT GAT ACG GCG ACC ACC GAG ATC TAC ACX XXX XXX XAC ACT CTT TCC CTA CAC GAC GCT CTT CCG ATC T–3′) and 2nd-PCR-reverse (5′–CAA GCA GAA GAC GGC ATA CGA GAT XXX XXX XXG TGA CTG GAG TTC AGA CGT GTG CTC TTC CGA TCT–3′). A 2nd PCR blank (2B) was made during this process.

To monitor contamination during on-site filtration, DNA extraction, and 1st and 2nd PCRs, 321 blanks were made (FB = 191, EB = 52, 1B = 65, 2B = 13) and subjected to the library preparation procedure. They were divided into four sets and the libraries were pooled in equal volumes into four 1.5-ml tubes. The target amplicons (ca. 370 bp) were purified using a 2% E-Gel Size Select agarose gel (Invitrogen, Carlsbad, CA). The concentration of the size-selected libraries was measured using a Qubit dsDNA HS Assay Kit and a Qubit fluorometer (Life Technologies, Carlsbad, CA), diluted to 12.0 pM with HT1 buffer (Illumina), and sequenced on the MiSeq platform (Illumina, San Diego, CA) using a MiSeq v2 Reagent Kit, 300 cycles (Illumina) with a PhiX Control v3 (Illumina, San Diego, CA) spike-in (expected at 5%), following the manufacturer’s protocol. To remove residual contamination from the MiSeq flow path, the flow channel was washed with hypochlorous acid before each operation. All raw DNA sequence data and associated information were deposited in DDBJ/EMBL/GenBank (accession number DRA007474).

### Data preprocessing and taxonomic assignment

Data preprocessing and analyses were performed using MiSeq raw reads from the 528 samples. Using USEARCH ver. 10.0.240^45^, quality-filtered forward and reverse reads were merged, primer sequences were removed, low-quality reads were filtered, and reads were dereplicated and denoised to obtain amplicon sequence variants (ASVs). Finally, fish species (molecular operational taxonomic units; MOTUs) were assigned to ASVs with a sequence identity of > 80% with the reference sequences. A custom reference database (see details in Supplementary Information S1) was used. Four expert ichthyologists (M.M., H.Motomura, T.Sado, and H.S.) revised the taxonomic assignments based on phylogenetic relationships combined with their knowledge of species distributions and dominance. The details of data processing, database assembly, and taxonomic assignment are described in Supplementary Information S1.

To focus on coastal fishes, all fishes principally inhabiting regions outside of the coastal areas (deep-sea fishes, oceanic epipelagic fishes, pure freshwater fishes, and all non-native fishes originating from food materials and carryover contamination) were excluded (Supplementary Table S2). Read counts were subsequently rarefied to the approximate minimum numbers (Supplementary Table S3).

### Collecting environmental covariates

To reduce the burden of fieldwork, only seawater temperature and salinity were measured at each site after water sampling and on-site filtration. Additionally, four environmental covariates were collected from publicly available satellite data^46^: annual minimum and maximum sea surface temperatures in 2017, mean chlorophyll a concentration in June-August 2017 and mean particulate inorganic carbon concentration in June-August 2017. The resolution of all collected satellite data is 4 km × 4 km. The field-measured seawater temperature and salinity were missing at ten sites due to instrument failure. Therefore, data from these sites were removed from the subsequent analysis. Furthermore, the following MOTU data were removed: (1) MOTUs categorised as species complex (i.e. including several species) and (2) MOTUs occurring at fewer than six sites. Consequently, the subsequent analyses included 519 species from 518 sampling sites.

### Nonlinear multivariate analysis

To better understand the regional community structure, sampling sites were geographically grouped into eight districts based on commonly used climate classifications and geographic proximity: Hokkaido Island (HKD, *n* = 71), East Main Island along the Pacific Ocean (EMP, *n* = 39), East Main Island along the Japan Sea (EMJ, *n* = 39), West Main Island along the Pacific Ocean (WMP, *n* = 95), West Main Island along the Japan Sea (WMJ, *n* = 90), West Main Island along inland sea (WMI, *n* = 63), Izu-Ogasawara Island (IOI, *n* = 51) and Satsuma-Ryukyu Island (SRI, *n* = 80). A nonlinear dimensionality reduction method, t-distributed stochastic neighbor embedding (t-SNE)^47^, was used to capture the structures of the entire regional fish community based on Jaccard distances between 518 sites.

### Generalised linear latent variable model

Joint species distribution models are used to analyse co-occurrence patterns while accounting for missing predictors. In this study, a generalised linear latent variable model (GLLVM)^7–9^ with spatially autocorrelated latent variables (LVs) was used as a joint species distribution model to explore the hidden niche space. The GLLVM decomposes high-dimensional residual correlations using low-dimensional LVs, thus allowing fitting to relatively large datasets. The model structure was a logistic regression with environmental covariates ($\:{\boldsymbol{x}}_{\boldsymbol{i}}$) and spatially autocorrelated LVs ($\:{\boldsymbol{u}}_{\boldsymbol{i}}$) as explanatory variables and species presence-absence ($\:{y}_{ij}$) as response variables:

$$\:{y}_{ij}\:\sim\:\mathrm{B}\mathrm{e}\mathrm{r}\mathrm{n}\mathrm{o}\mathrm{u}\mathrm{l}\mathrm{l}\mathrm{i}\left({p}_{ij}\right),\:\mathrm{l}\mathrm{o}\mathrm{g}\mathrm{i}\mathrm{t}\left({p}_{ij}\right)={\beta\:}_{j0}+{\boldsymbol{x}}_{i}^{\top\:}\:{\boldsymbol{\beta\:}}_{j}+{\boldsymbol{u}}_{i}^{\top\:}\:{\boldsymbol{\gamma\:}}_{j}+{\alpha\:}_{i},$$

for fish species $\:j$ = 1, …, 519 at sampling site $\:i$ = 1, …, 518. $\:{\boldsymbol{\beta\:}}_{j}$ and $\:{\boldsymbol{\gamma\:}}_{j}$ are species-specific responses related to the environmental covariates and LVs, respectively. $\:{\beta\:}_{j0}$ is the species-specific intercept and $\:{\alpha\:}_{i}$ is random site effects. The random site effects can mitigate the bias owing to the variation in total eDNA concentrations among sites^9^. The spatial autocorrelation was modelled by Gaussian Markov random field^48^ (Supplementary Figure S4). The details of the model structure are described in Supplementary Information S2.

This study considered three candidate models with different sets of environmental covariates. As environmental covariates, Model (a) included the two field-measured variables, Model (b) included the four satellite variables and Model (c) included both the two field-measured variables and the four satellite variables. Furthermore, models with different numbers of latent variables (from 0 to 5) were also considered for each candidate model. Using five-fold cross validation with negative log-likelihoods as the validation loss, the Model (a) with three spatially autocorrelated LVs was selected as the best-fit model from 18 candidate models. In the main text, the results of this best-fit model were mainly reported for simplicity, whereas the results of the best-fit model of Models (b–c) were reported in Supplementary Information S2. Model (a) exhibited considerably higher model predictive ability for the regional fish community than Models (b–c) (Supplementary Table S5). Nevertheless, these models estimated similar geological variations in latent variables 1 and 2 and reproduced the five hypothetical biogeographic boundaries described in the following paragraph (Supplementary Figure S5).

As several previous studies^49,50^ did, our study defined biogeographic boundaries based on the ordination space (Fig. 2). Given that GLLVM directly relates ordination axes (i.e. latent variables) to species occurrence probabilities, community compositions are expected to change largely at the boundary of the zero-value line. Thus, zero-value lines were used to identify hypothetical biogeographic boundaries. The first and second LVs revealed five hypothetical boundaries between distinct local communities (Fig. 2 and Supplementary Figure S2). The third LV showed broad zero-value areas (not zero-value lines), suggesting no boundaries between communities (Supplementary Figure S2). Species niche centres were defined as the position with the highest occurrence probability in the niche space. For this, species occurrence records in geographic space were mapped onto the niche space and then the occurrence probability on the niche space was calculated using a kernel density estimation method.

### Calculation of response diversity

To calculate the response diversity of a local community to each LV, 100 communities were randomly generated with estimated occurrence probabilities ($\:\boldsymbol{p}$). The interquartile range of the response to each LV ($\:\boldsymbol{\gamma\:}$) was calculated for the species comprising each generated community and the interquartile ranges over all 100 generated communities were averaged as the expected response diversity. The interquartile range was used instead of the other dispersion proxies (e.g. variance) to reduce the influence of outliers due to estimation error.

Spectral clustering and linear regression were used to investigate the relationship between species richness and response diversity. Local communities were classified into high and low expected response diversity using spectral clustering (the number of clusters was set to two). Then, linear regression was applied to each cluster:

$$\:\mathrm{l}\mathrm{o}\mathrm{g}\left(response\:diversity\right)\:\sim\:\mathrm{l}\mathrm{o}\mathrm{g}\left(richness\right).$$

To ensure homogeneity of variances, both species richness and response diversity were log-transformed. Because spectral clustering can produce several different results due to the randomness in the optimisation process, clustering was repeated 100 times and finally the clustering result with the highest log-likelihood was selected for linear regressions. All codes are available at https://doi.org/10.5281/zenodo.17823192.

## Supplementary Information

Below is the link to the electronic supplementary material.

Supplementary Material 1

Supplementary Material 2

## Data Availability

Data are available on Supplementary Table S1-S4 and will be deposited in ANEMONE DB (https://db.anemone.bio/). All raw DNA sequence data and associated information are deposited in DDBJ/EMBL/GenBank (accession number DRA007474). The custom reference database is available on https://doi.org/10.5281/zenodo.17823192.
