# Supplementary material for: Large-scale environmental DNA survey reveals niche axes of a regional coastal fish community
Source: Sci Rep. 2026 Feb 16;16:3276. doi: 10.1038/s41598-025-31307-4 (PMC12909844; doi:10.1038/s41598-025-31307-4)
Supplement: Supplementary file 2 — Supplementary Material 2 [file 41598_2025_31307_MOESM2_ESM.pdf]

Supplementary Information Materials for

Large-scale environmental DNA survey reveals niche axes of a regional coastal fish community

Yutaka Osada, Masaki Miya, Hitoshi Araki, Hideyuki Doi, Akihide Kasai, Reiji Masuda, Toshifumi Minamoto, Satoquo Seino, Teruhiko Takahara, Satoshi Yamamoto, Hiroki Yamanaka, Mitsuhiro Aizu-Hirano, Keiichi Fukaya, Takehiko Fukuchi, Ryo O. Gotoh, Masakazu Hori, Midori Iida, Tomohito Imaizumi, Tadashi Kajita, Takashi Kanbe, Tanaka Kenta, Yumi Kobayashi, Tomohiko Matsuura, Hiroki Mizumoto, Hiroyuki Motomura, Hiroaki Murakami, Shin-ichiro Oka, Tetsuya Sado, Hiroshi Senou, Koichi Shibukawa, Tomoki Sunobe, Hiroshi Takahashi, Koji Takayama, Kenji Nohara, Katsuhiko Tanaka, Hisashi Yamakawa, Satoru Yokoyama, Seokjin Yoon, Michio Kondoh

This PDF file includes:

S1. Environmental DNA sample collection and analysis

1. Water sampling and on-site filtration
2. DNA extraction
3. Paired-end library preparation and sequencing
4. Data preprocessing and taxonomic assignment

Tables S1–S4

S2. GLLVM analysis

1. Model structure
2. Application and results

Table S5–S6, Figures S1–S5

S1. Environmental DNA sample collection and analysis

1. Water sampling and on-site filtration

Seawater samples were obtained at 528 sites during the summer of 2017, from June 5 to August 18 (Supplementary Table S1). A low-tech bucket-sampling technique was employed to collect seawater using a folding 7.8-l polypropylene bucket (Soft Bucket 8; ISETO, Osaka, Japan) fastened to a 15 m vinylon rope of 6 mm in diameter. Before and during water sampling, disposable gloves were worn on both hands and two sets of on-site filtration kits consisting of a Sterivex filter cartridge (pore size 0.45 µm; Merck Millipore, Billerica, MA) and a 50-ml disposable syringe with a luer lock connector (Terumo Corp., Tokyo, Japan) were assembled. Then, the bucket inside and the tip of the rope were thoroughly decontaminated with a foam-style 10% bleach solution and the equipment was brought to the sampling point. Surface seawater was collected by casting and retrieving the bucket full of seawater. This collection procedure was repeated 10 times to minimise sampling bias at each site.

On-site filtration was performed using two pairs of the filtration kit (filter cartridge and syringe) by two researchers to obtain duplicate samples. For the collection of each seawater sample, the filter cartridge was removed from the syringe, approximately 50 ml of seawater was drawn into the syringe by pulling the plunger, the filter cartridge was reattached to the syringe and the plunger was pushed for filtration. This step was repeated twice for each cast of the bucket and the final filtration volume reached 1,000 ml × 2 with 10 casts of the bucket. When the filter clogged before reaching 1,000 ml, the total volume of water filtered was recorded (200–900 ml from 28 sites, mostly from brackish or turbid waters around the big cities). After on-site filtration, an outlet port of the filter cartridge was sealed with Parafilm (LMS, Tokyo, Japan), 1.6 ml of RNA*later* (Thermo Fisher Scientific, Waltham, MA) was added to the cartridge from an inlet port using a disposable capillary pipette (As One Corp., Osaka, Japan) to prevent eDNA degradation, and the inlet port was sealed with either the film or a cap for preservation. A filtration blank (FB) was prepared by filtering 500 ml of purified water in the same manner at the end of each water-sampling day. The filtered cartridges were transported to the laboratory in a portable cooler with ice packs and kept at −20°C in the freezer until eDNA extraction.

2. DNA extraction

The workspace and equipment were thoroughly sterilised with hypochlorite solution before DNA extraction. Low-retention filtered pipette tips and microtubes were used to conduct all eDNA extraction manipulations. The eDNA extraction room was physically separated from pre- and post-PCR rooms to safeguard against carryover contamination from PCR products. eDNA was extracted from the filter cartridges using a DNeasy Blood & Tissue Kit (Qiagen, Hilden, Germany) following

the methods developed by Miya et al. (2016), with slight modifications. An aspirator (Vac-Man Laboratory Vacuum Manifold and Welch Vacuum Pump; Promega, Madison, WI) was used in a UV-sterilised cabinet to remove redundant seawater and RNA*later* from the cartridge. The filter cartridge was subjected to lysis using proteinase K. Before lysis, PBS (220 µl), Proteinase K (20 µl), and Buffer AL (200 µl), included in the kit, were mixed. An outlet port of the filter cartridge was sealed in advance, and the mixed solution was gently pipetted into the cartridge from its inlet port. After the inlet port was sealed, the cartridge was placed in a 56°C preheated incubator for 10 min while the cartridge was stirred using a rotator (RotoFlex Plus; Argos Technologies, Vernon Hills, IL) in oscillation mode. After incubation, the seal was removed from the inlet port and the port was connected to a 2 ml tube (DNA LoBind tube; Eppendorf Corp., Hamburg, Germany) for DNA collection. The combined unit was placed in a 50 ml conical tube and the capped tube was centrifuged at 6,000 *g* for 1 min to collect the crude DNA extract. Following the manufacturer's protocol, the collected DNA extract was purified using the kit and the final elution volume was set at 200 µl. During this process, an extraction blank (EB) was made using fresh Milli-Q Water and a new Sterivex filter cartridge.

3. Paired-end library preparation and sequencing

The workspace and equipment were thoroughly sterilized in a pre-PCR area with hypochlorite solution before library preparation. Low-retention filtered pipette tips and microtubes were used. Pre- and post-PCR manipulations were performed in two different dedicated rooms to safeguard against carryover contamination. Two-step PCR was employed for paired-end next-generation sequencer (NGS) library preparation by following previously developed methods (Miya et al. 2015, Minamoto et al. 2021). For the first-round PCR (1st PCR), a mixture of the two primer pairs was used: MiFish-U-forward (5′–ACA CTC TTT CCC TAC ACG ACG CTC TTC CGA TCT NNN NNN GTC GGT AAA ACT CGT GCC AGC–3′), MiFish-U-reverse (5′–GTG ACT GGA GTT CAG ACG TGT GCT CTT CCG ATC TNN NNN NCA TAG TGG GGT ATC TAA TCC CAG TTT G–3′), MiFish-E-forward-v2 (5′–ACA CTC TTT CCC TAC ACG ACG CTC TTC CGA TCT NNN NNN RGT TGG TAA ATC TCG TGC CAG C–3′) and MiFish-E-reverse-v2 (5′–GTG ACT GGA GTT CAG ACG TGT GCT CTT CCG ATC TNN NNN NGC ATA GTG GGG TAT CTA ATC CTA GTT TG–3′). These primer pairs co-amplify a hypervariable region of the fish mitochondrial 12S rRNA gene (ca. 172 bp; hereafter called the "MiFish sequence") and append primer-binding sites (5′ ends of the sequences before six Ns) for sequencing at both ends of the amplicon. Six random bases (Ns) in the middle of these primers were used to enhance cluster separation on the flow cells during initial base call calibration on the NGS platform.

The 1st PCR was carried out in a 12-µl reaction volume containing 6.0 µl of 2× KAPA HiFi HotStart ReadyMix (Kapa Biosystems, Wilmington, MA), 2.8 µl of a mixture of the two MiFish primer pairs in equal volumes (U/E forward and reverse primers; 5 µM), 1.2 µl of sterile distilled H_2O, and a 2.0-µl eDNA template (a mixture of the duplicated eDNA extracts in equal volumes). To minimise PCR dropout during the 1st PCR, eight replications were performed for the same eDNA template using a strip of eight tubes (0.2 ml). The thermal cycle profile after an initial 2-min denaturation at 95°C was as follows: denaturation at 98°C for 20 sec, annealing at 65°C for 15 sec, and extension at 72°C for 15 sec, 35 cycles, with a final extension at the same temperature for 5 min. A 1st PCR blank (1B) was prepared in addition to FB and EB during this process. Note that only a single tube was used for each type of blank (FB, EB, and 1B) to reduce the cost of the experiments. After completing of the 1st PCR, equal volumes of the PCR products were pooled from the eight replications in a single tube and purified using a GeneRead Size Selection Kit (Qiagen, Hilden, Germany). Subsequently, the purified target products (ca. 300 bp) were quantified using TapeStation 2200 (Agilent Technologies, Tokyo, Japan) and diluted to 0.1 ng/µl using Milli Q water, and the diluted products were used as templates for the second-round PCR (2nd PCR). For the three types of blanks (FB, EB, and 1B), the 1st PCR products were purified in the same manner, but the purified PCR products were not quantified. Samples were diluted at an average dilution ratio for positive samples and used as templates for the 2nd PCR. The following two primers were used for the 2nd PCR to append dual-indexed sequences (eight nucleotides indicated by Xs) and flow cell-binding sites for the MiSeq platform (5′ ends of the sequences before eight Xs): 2nd-PCR-forward (5′–AAT GAT ACG GCG ACC ACC GAG ATC TAC ACX XXX XXX XAC ACT CTT TCC CTA CAC GAC GCT CTT CCG ATC T–3′) and 2nd-PCR-reverse (5′–CAA GCA GAA GAC GGC ATA CGA GAT XXX XXX XXG TGA CTG GAG TTC AGA CGT GTG CTC TTC CGA TCT–3′). The 2nd PCR was carried out in a 12-µl reaction volume containing 6 µl of 2 × KAPA HiFi HotStart ReadyMix, 0.7 µl of each primer (5 µM), 3.8 µl of sterile distilled H_2O, and 1.5 µl of template (0.1 ng/µl each, with the exception of blanks). The thermal cycle profile after an initial 2 min of denaturation at 95°C was as follows: denaturation at 98°C for 20 sec, annealing and extension combined at 72°C (shuttle PCR) for 15 sec, 10 cycles, with a final extension at the same temperature for 5 min. A 2nd PCR blank (2B) was made during this process in addition to FB, EB, and 1B.

To monitor contamination during on-site filtration, subsequent DNA extraction, and 1st and 2nd PCRs of the 528 samples, 321 blanks (FB = 191, EB = 52, 1B = 65, and 2B = 13) were made and then subjected to the above library preparation procedure. The 849 dual-indexed libraries (528 samples and 321 blanks) were divided into four sets (sets A–D) and each of the individual libraries from the four sets in equal volumes were pooled into four 1.5 ml tubes. Then, the four pooled dual-

indexed libraries were electrophoresed using a 2% E-Gel Size Select agarose gel (Invitrogen, Carlsbad, CA) and the target amplicons (ca. 370 bp) were excised by retrieving them from the recovery wells using a micropipette. The concentration of the size-selected libraries was measured using a Qubit dsDNA HS Assay Kit and a Qubit Fluorometer (Life Technologies, Carlsbad, CA), diluted to 12.0 pM with HT1 buffer (Illumina) and sequencing on the MiSeq platform (Illumina, San Diego, CA) using a MiSeq v2 Reagent Kit, 300 cycles (Illumina) with a PhiX Control v3 (Illumina, San Diego, CA) spike-in (expected at 5%) following the manufacturer's protocol. To remove residual contamination from the MiSeq flow path, the flow channel was washed with hypochlorous acid before each operation. All raw DNA sequence data and associated information are deposited in DDBJ/EMBL/GenBank (accession number DRA007474).

4. Data preprocessing and taxonomic assignment

Data preprocessing and analyses of MiSeq raw reads from the 528 samples were performed using USEARCH v10.0.240 (Edgar 2010). All 321 blank samples were preprocessed and analysed separately from the 528 samples following the methods described below. (1) Forward (R1) and reverse (R2) reads were merged by aligning the two reads using the *fastq mergepairs* command. During this process, low-quality tail reads with a Phred score of <10, short reads (<100 bp) after tail trimming, and paired reads with differences at >5 positions in the aligned region (ca. 65 bp) were discarded. (2) Primer sequences were removed from merged reads using the *fastx truncate* command. (3) Reads without primer sequences underwent quality filtering using the *fastq filter* command to remove low-quality reads with an expected error rate of >1% and short reads of <120 bp. (4) The preprocessed reads were dereplicated using the *fastx uniques* command and all singletons, doubletons and tripletons were removed from subsequent analyses to avoid false positives (Edgar 2010). (5) The dereplicated reads without single to tripletons were denoised using the *unoise3* command to generate amplicon sequence variants (ASVs) without putatively chimeric and erroneous sequences (Callahan et al. 2017). (6) ASVs were used for taxonomic assignments to the species level (molecular operational taxonomic units; MOTUs) using the *usearch global* command with a sequence identity threshold of >98.5% with the reference sequences (two nucleotide differences allowed) and a query coverage of ≥90%. For ASVs with sequence identities of 80%–98.5%, "U98.5" was added before the corresponding species (e.g. U98.5 *Pagrus major*), followed by clustering at the 0.985 level using the *cluster smallmem* command. An incomplete reference database necessitates this clustering step for the detection of multiple MOTUs with identical species names. Multiple MOTUs were annotated as 'gotu1, 2, 3…' and tabulated all of these outputs (MOTUs plus U98.5 MOTUs) with read abundances. ASVs with sequence identities of <80% (saved as 'no hit') were excluded

from the above taxonomic assignments and downstream analyses because they were all non-fish organisms.

To focus on coastal fish species alone, all fishes principally inhabiting areas outside of coastal areas within the reaches of the bucket sampling were removed: deep-sea fishes (e.g. lanternfishes in the family Myctophidae), oceanic epipelagic fishes (e.g. tunas in the family Scombridae), and pure freshwater fishes (e.g. nearly all members of the family Cyprinidae) as well as all non-native fishes putatively originating from food materials (Supplementary Table S2). Species probably originating from carryover contamination (e.g. Southeast Asian freshwater fishes) were also removed to avoid false positives; however, such contamination was considered to be minor because we detected only a few reads (11.2 reads on average) from 321 blank samples, compared with an average of 30,608 reads from 528 samples. The ratio (11.2/30,608 reads, 0.036%) was used to determine the number of reads removed from a rarefied list of fishes (see below).

For constructing a custom reference database, MiFish sequences were assembled from 5,691 fish species in the M.M. lab. In addition, all fish whole-mitochondrial genomes and 12S rRNA gene sequences were downloaded from NCBI as of June 26, 2017, and MiFish sequences were extracted using a custom Perl script (Miya et al. 2015). MiFish sequences were combined from the two sources in FASTA format, and the combined sequences were used as the custom reference database for taxonomic assignments. The final reference database consisted of 14,650 sequences from 7,555 species belonging to 2,612 genera and 464 families. The automatic taxonomic assignments were refined based on family-level phylogenies reproduced from MOTUs, U98.5 MOTUs, and the reference sequences. For each family, representative sequences (most abundant reads) were assembled from MOTUs and U98.5 MOTUs and all reference sequences from the family and an outgroup (a single sequence from the closely related family) were added in FASTA format. The FASTA file was used to generate a multiple alignment using MAFFT (Katoh and Toh 2008) with default parameter settings. A neighbor-joining (NJ) tree was constructed based on the aligned sequences using MEGA7 (Kumar et al. 2016) with the Kimura two-parameter model. Distances were calculated using pairwise deletion of gaps with gamma-distributed variation among sites (shape parameter = 1). Bootstrap resampling (n = 100) was performed to estimate statistical support for internal branches and the tree was rooted with the outgroup. Finally, four expert ichthyologists (M.M., H.Motomura, T.Sado and H.S.) revised the taxonomic assignments and removed problematic MOTUs based on phylogenetic relationships, combined with their prior knowledge of species distribution and dominance. For U98.5 MOTUs placed within a monophyletic group consisting of a single genus, the genus was assigned to unidentified MOTUs with “sp” plus sequential numbers (e.g. *Pagrus* sp1, sp2, sp3…). The remaining MOTUs ambiguously placed in the family-level tree were

labelled with the family name and 'sp' plus sequential numbers (e.g. Sparidae sp1, sp2, sp3…). Based on the revised taxonomic assignment, a list of fishes was constructed with the denoised read numbers (average number of reads per sample = 30,608) (Supplementary Table S3). Scientific names were updated based on the most recent Japanese fish species list (Motomura 2023). The read numbers were fixed to the approximate rarefied minimum numbers (10,000; range 9,188–102,234) and taxa with reads <5 were removed from subsequent analyses (Supplementary Table S4). The custom reference database and detailed information on the taxonomic assignment of each MOTU are available by contacting M.M. (masaki_miya@me.com).

Supplementary Tables S1-S4 | The tables below are provided as an external .xlsx file.

Table S1 | List of 528 sampling site.

Table S2 | List of 224 non-coastal fish species detected for eDNA samples. These species were excluded from community analyses.

Table S3 | List of 1,220 coastal fish species detected for eDNA samples and their representative sequences.

Table S4 | Read counts for 1,220 coastal fish species at 528 sampling sites with rarefaction at 10,000 reads and taxa with <5 reads were excluded from subsequent analyses.

S2. GLLVM analysis

1. Model structure

Joint species distribution models are promising tools for analysing species co-occurrence patterns while accounting for missing predictors (Warton et al. 2015, Poggiato et al. 2021). This study used a generalised linear latent variable model (GLLVM; Thorson et al. 2015, Ovaskainen et al. 2016, Niku et al. 2019) with spatially-autocorrelated latent variables as a joint species distribution model to explore the hidden niche space of community structure. The GLLVM decomposes high-dimensional residual correlations using low-dimensional latent variables, thus allowing fitting to relatively large datasets. Consider a sample of presence-absence y_{ij} for species $j = 1, \dots, M$ at sampling site $i = 1, \dots, N$. The GLLVM regresses the species occurrence probability against K environmental covariates $\boldsymbol{x}_i = (x_{i1}, \dots, x_{iK})^\top$ and F latent variables $\boldsymbol{u}_i = (u_{i1}, \dots, u_{iF})^\top$ as follows:

$$y_{ij} \sim \text{Bernoulli}(p_{ij}), \quad \text{logit}(p_{ij}) = \Lambda_{ij} = \beta_{j0} + \boldsymbol{x}_i^\top \boldsymbol{\beta}_j + \boldsymbol{u}_i^\top \boldsymbol{\gamma}_j + \alpha_i \qquad \text{(S1)},$$

where $\boldsymbol{\beta}_j = (\beta_{j1}, \dots, \beta_{jK})^\top$ and $\boldsymbol{\gamma}_j = (\gamma_{j1}, \dots, \gamma_{jF})^\top$ are species-specific responses related to the environmental covariates and latent variables, respectively. β_{j0} is the species-specific intercept and

α_i is random site effects. The random site effects can mitigate the bias owing to the variations of total eDNA concentrations among sites (Niku et al. 2019). With reference to previous studies (Thorson et al. 2015), triangular constraints were imposed into species-specific coefficients to ensure their identifiability:

$$\boldsymbol{\gamma} = \begin{bmatrix} \gamma_{11} & 0 & 0 & \cdots & 0 \\ \gamma_{21} & \gamma_{22} & 0 & \cdots & 0 \\ \gamma_{31} & \gamma_{31} & \gamma_{33} & \ddots & 0 \\ \vdots & \vdots & \vdots & \ddots & \vdots \\ \gamma_{M1} & \gamma_{M2} & \gamma_{M3} & \cdots & \gamma_{MF} \end{bmatrix} \quad \text{(S2).}$$

Latent variables ($\boldsymbol{u}$) is $N \times F$ matrix, and their columns are independently generated by multivariate normal distribution with zero means and arbitrary covariance matrices. In this study, to model spatial autocorrelation between sampling sites with Gaussian Markov random field (GMRF; Lindgren et al. 2011), the covariance matrices were specified by Matérn covariance with scaling parameter (κ) and unit marginal variance. A triangulated adjacent map was constructed to define the adjacency of sampling sites for the GMRF (Lindgren and Rue 2015, Krainski et al. 2018; Supplementary Figure S1). For model fitting, Template Model Builder (Kristensen et al. 2016) was used, wherein which the marginal likelihood of $\boldsymbol{\alpha}$ and $\boldsymbol{u}$ were evaluated as random effects by Laplace approximation.

2. Application and results

When applied to our regional fish community data, this study considered three candidate model with different sets of environmental covariates. As environmental covariates, Model (a) included two field-measured variables (temperature and salinity), Model (b) included four satellite variables (annual minimum and maximum sea surface temperatures, chlorophyll a concentration and particulate inorganic carbon concentration) and Model (c) included both the two field-measured variables and the four satellite variables. Furthermore, candidate models with different numbers of latent variables (from 0 to 5) were also considered for each candidate model. Before model fitting, several data were removed when (1) environmental data were missing due to instrument failure, (2) fish categorisation was species complex (i.e. including several species) and (3) fish species occurred at fewer than six sites. As a result, our model targeted 519 species from 518 sampling sites.

To avoid model overfitting, 18 candidate models were compared using five-fold cross validation with negative log-likelihoods as validation loss (Supplementary Table S5). The cross validation showed that the best-fit model is the Model (a) with three spatially autocorrelated latent variables (hereafter, model A3). Thus, the results of model A3 are discussed in the main text. For Models (b) and (c), the model with two spatially autocorrelated latent variables was selected as the best-fit

model. As these two best-fit models produced similar results (Supplementary Table S6), we report the results of the best-fit model in Model (c) (hereafter, model C2) here.

The results showed that in model C2, environmental covariates explained 80.1% of the variation in fish biodiversity, while latent variables explained the remaining 19.9% (Supplementary Table S6). In particular, annual minimum sea surface temperature and chlorophyll a concentration explained most of this variation (47.7% and 20.9%, respectively), followed by annual maximum temperature (6.9%), particulate inorganic carbon concentration (2.0%), field-measured temperature (1.6%) and field-measured salinity (1.0%). Model C2 could reproduce latent variables 1 and 2 similar to model A3, but not the latent variable 3. The latent variables 1 and 2 explained 3.0% and 16.9% of the variation, respectively. Compared to model A3, the explained variation of latent variable 1 decreased considerably (from 58.9% to 3.0%), probably because the spatial pattern of the latent variable 1 was largely explained by the spatial pattern of annual minimum temperature, except for Satsuma-Ryukyu Islands. As well as the latent variable 1, the latent variable 3 of model A3 appears to be explained by chlorophyll a concentration, as both are associated with enclosed inland seas. Thus, the result of model C2 suggests that long-term environmental factors (e.g., satellite data) may be more important for the regional fish community than environmental factors at the time of sampling and that using appropriate environmental covariates can reduce the explained variation of latent variables. By comparing cross validation with models A3 and C2 and checking estimates of species-specific responses of each covariate, we found that the estimation of model C2 was affected by overfitting problems. Therefore, the results of model C2 should be interpreted with caution because overfitted species-specific responses inflate the explained variation of its related covariate. Interestingly, models A3 and C2 produced similar estimates of the geological variation in latent variables 1 and 2 (Figure 2 and Supplementary Figure S5), despite the significant difference in the variations they explained. Five hypothetical biogeographic boundaries between distinct local communities were also reproduced (Supplementary Figure S5, B1–B5; see Methods for boundary identification).

References

1. Miya, M. et al. Use of a filter cartridge for filtration of water samples and extraction of environmental DNA. *J. Visual. Exper* 117, 5474 (2016).
2. Miya, M. et al. MiFish, a set of universal PCR primers for metabarcoding environmental DNA from fishes: detection of more than 230 subtropical marine species. *R. Soc. Open Sci.* 2, 150088 (2015).
3. Minamoto, T. et al. An illustrated manual for environmental DNA research: water sampling guidelines and experimental protocols. *Environ. DNA* 3: 8-13 (2021)
4. Edgar, R. C. Search and clustering orders of magnitude faster than BLAST. *Bioinformatics* 26, 2460–2461 (2010).
5. Callahan, B. J., McMurdie, P. J. & Holmes, S. P. Exact sequence variants should replace operational taxonomic units in marker-gene data analysis. The ISME Journal 11, 2639–2643 (2017).
6. Katoh, K. & Toh, H. Recent developments in the MAFFT multiple sequence alignment program. *Brief Bioinformatics* 9, 286–298 (2008).
7. Motomura, H. *List of Japan's all fish species. Current standard Japanese and scientific names of all fish species recorded from Japanese waters.* Online ver. 23. https://www.museum.kagoshima-u.ac.jp/staff/motomura/jaf.html (2023).
8. Kumar, S., Stecher, G. & Tamura, K. MEGA7: molecular evolutionary genetics analysis version 7.0 for bigger datasets. *Mol. Biol. Evol.* 33, 1870–1874 (2016).
9. Warton, D. I. et al. So many variables: joint modeling in community ecology. *Trend. Ecol. Evol.* 30: 766-779 (2015).
10. Poggiato, G. et al. On the interpretations of joint modeling in community ecology. *Trend. Ecol. Evol.* 36: 391-401 (2021).
11. Thorson, J. T. et al. Spatial factor analysis: a new tool for estimating joint species distributions and correlations in species range. *Methods Ecol. Evol.* 6: 627-637 (2015).
12. Ovaskainen, O. et al. Uncovering hidden spatial structure in species communities with spatially explicit joint species distribution models. *Methods Ecol. Evol.* 7: 428-436 (2016).
13. Niku J. et al. gllvm: Fast analysis of multivariate abundance data with generalized linear latent variable models in R. *Methods Ecol. Evol.* 10: 2173-2182 (2019).
14. Lindgren, F., Rue, H. & Lindström, J. An explicit link between Gaussian fields and Gaussian Markov random fields: the stochastic partial differential equation approach. *J. R. Statist. Soc. B* **73**: 423-498 (2011).
15. Lindgren, F., Rue, H. Bayesian spatial modelling with R-INLA. *J. Stat. Software* **63**, 1-25 (2015)

16. Krainski, E. T. et al. 2018. *Advanced spatial modeling with stochastic partial differential equations using R and INLA.* Chapman and Hall/CRC press. Boca Raton, FL.

17. Kristensen, K., Nielsen, A., Berg, C. W., Skaug, H., Bell, B. M. TMB: Automatic differentiation and Laplace approximation. *J. Stat. Software* **70**, 1–21 (2016)

Supplementary Table S5 | Training and validation loss for each candidate model. As environmental covariates, Model (a) included two field-measured variables (temperature and salinity), Model (b) included four satellite variables (annual minimum and maximum sea surface temperatures, chlorophyll a concentration and particulate inorganic carbon concentration) and Model (c) included both the two field-measured variables and the four satellite variables. For each candidate model, negative log-likelihood was calculated as model training loss and validation loss. Bold indicates the best candidate model with the lowest validation loss.

	(a) Field measured		(b) Satellite	
Number of latent variables	Training loss	Validation loss	Training loss	Validation loss
0	33063.8	10738.0	26487.0	16368.8
1	26510.9	13788.6	24380.4	13173.5
2	24435.5	9234.6	23010.5	**9156.8**
3	23018.4	**8069.7**	21964.0	9430.2
4	21885.1	8492.6	20983.6	9964.9
5	21103.3	9384.7	20201.9	11829.0

	(c) Field measured + Satellite	
Number of latent variables	Training loss	Validation loss
0	25128.1	16680.3
1	23355.9	14177.6
2	22083.5	**9541.5**
3	20971.8	10504.9
4	19978.9	12907.6
5	19181.0	15395.8

Supplementary Table S6 | The explained variances of environmental covariates and latent variables for the best-fit model of candidate Models (a–c).

	(a) Field measured	(b) Satellite	(c) Field measured + Satellite
SST (Field)	1.0%		1.6%
Salinity (Field)	0.7%		1.0%
Minimum SST		56.7%	47.7%
Maximum SST		7.2%	6.9%
Chlorophyll a conc.		16.2%	20.9%
PIC conc.		1.9%	2.0%
Latent variable 1	58.9%	2.1%	3.0%
Latent variable 2	37.4%	15.8%	16.9%
Latent variable 3	2.0%		
All environmental covariates	1.7%	82.0%	80.1%
All latent variables	98.3%	18.0%	19.9%

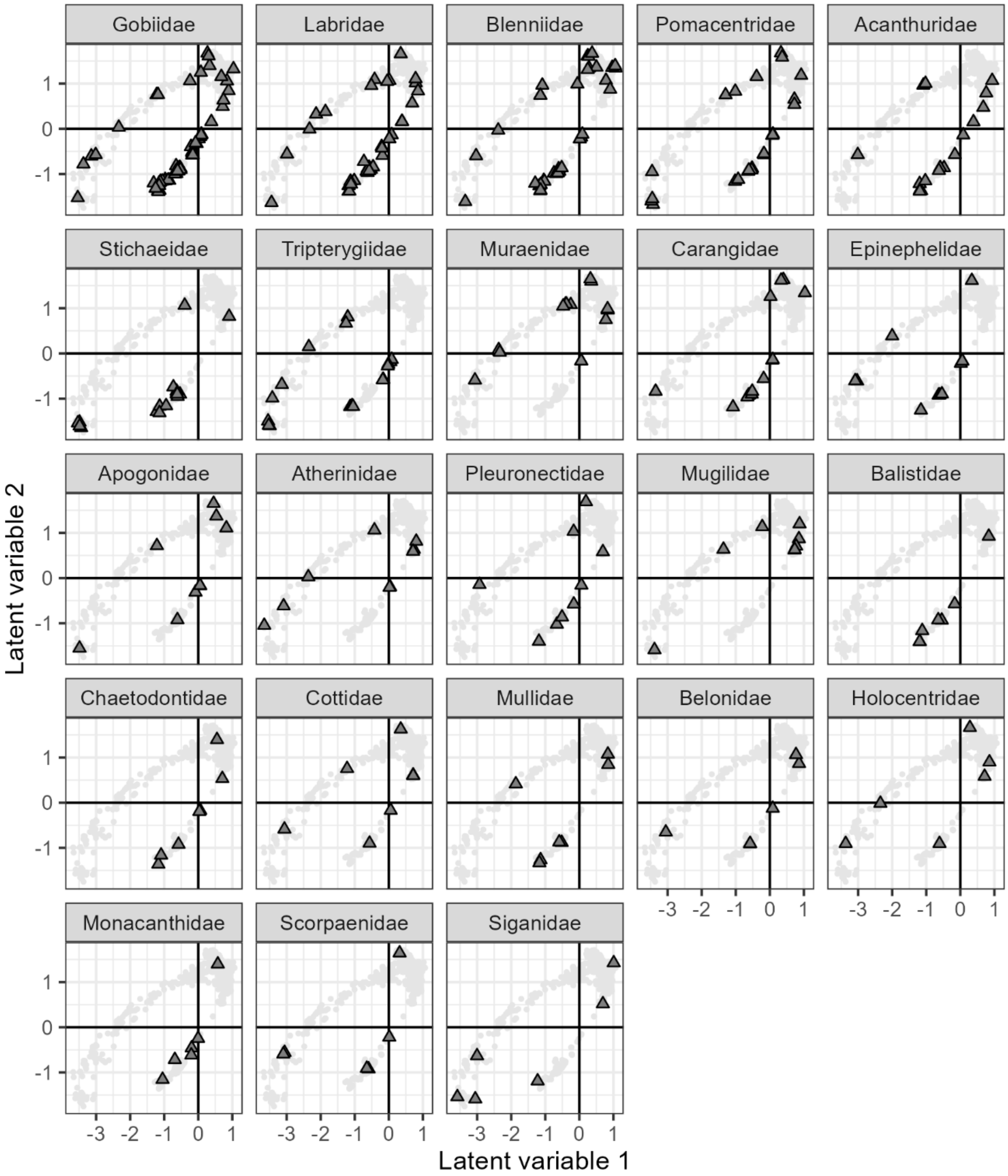

Supplementary Figure S1 | Niche space of representative families (including more than five species) with latent variables 1 and 2 as axes. Gray points represent the niche values for the sampling sites and triangles represent the niche centres of the analysed species.

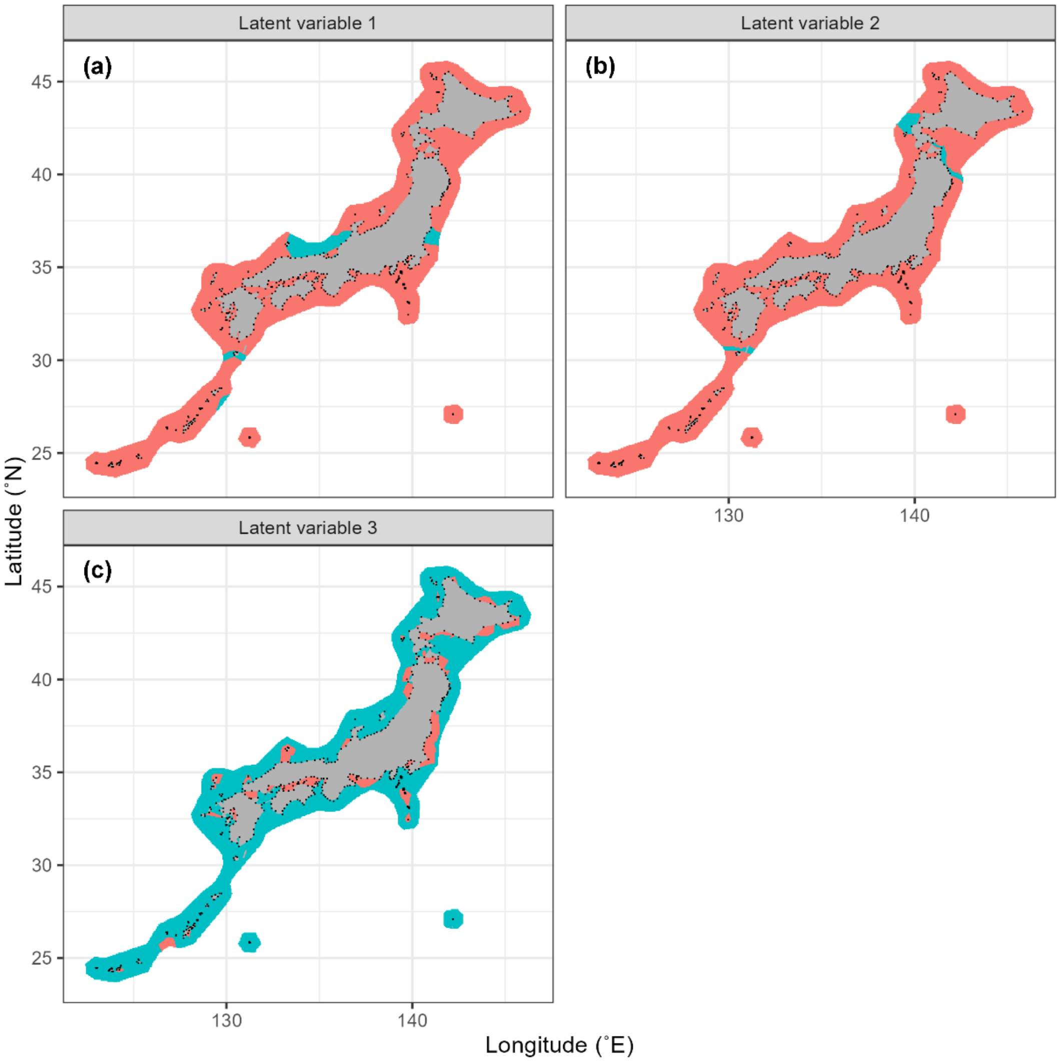

Supplementary Figure S2 | Near-zero area of latent variables. Blue and red areas represent the absolute value of latent variables that are less and greater than 0.1, respectively.

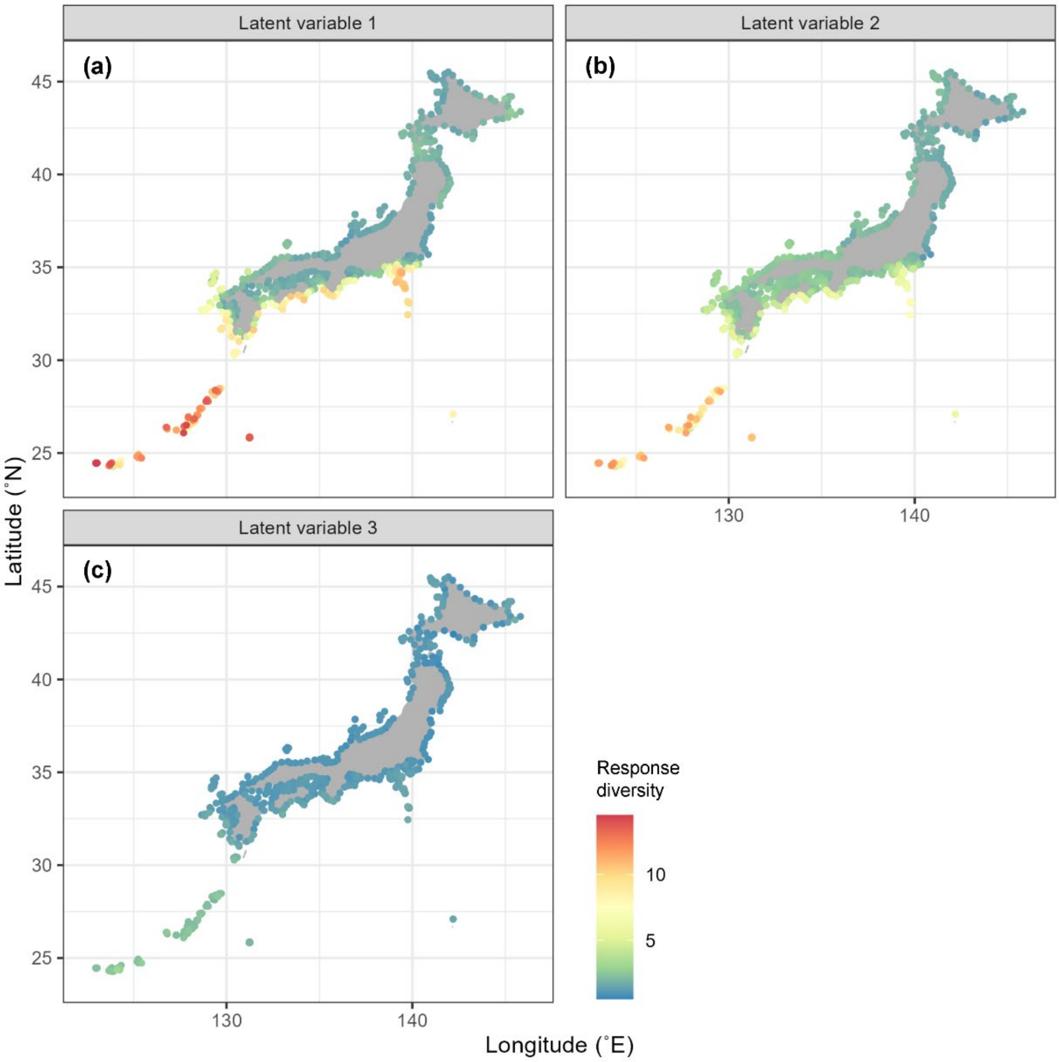

Supplementary Figure S3 | (a–c) Response diversity of local fish communities to each latent variable, calculated from the output of the generalised linear latent variable model. Each point represents a local community at each sampling site. Colour gradient from blue to red represents the response diversity from low to high.

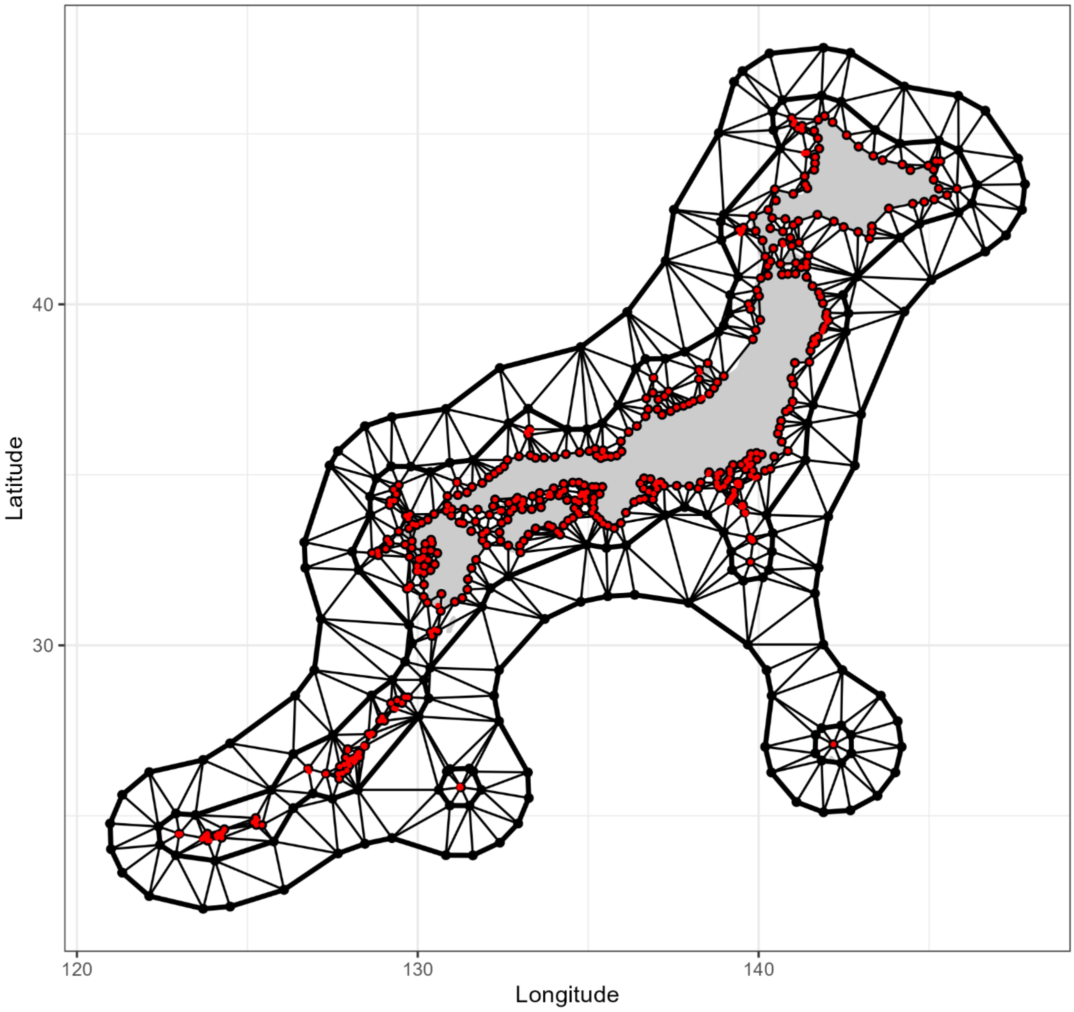

Supplementary Figure S4 | Triangulated adjacent map to model spatial autocorrelation between sampling sites for the Gaussian Markov random fields (GMRF) model. Points represent sampling sites (red) and additional boundary sites to improve the approximation of GMRF (black). Lines represent the adjacency of the sites, with bold lines representing the inner and outer model boundaries.

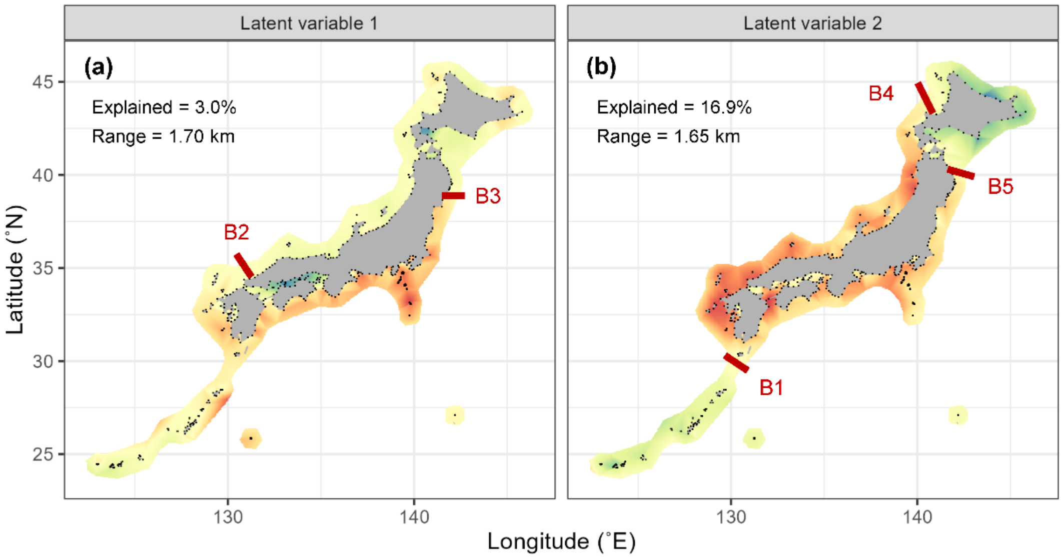

Supplementary Figure S5 | Geological variation in latent variables estimated as hidden niche axes from GLLVM model with both field-measured variables and satellite variables as covariates. Colour gradient from blue to red represents the values of scaled latent variables. Areas with similar colours exhibit similar niche values. Points represent sampling sites. Bold red lines represent five hypothetical boundaries between distinct local communities. The latent variables 1 and 2 explained 3.0% and 16.9% of the total variance, respectively. Range represents the scale of spatial autocorrelation obtained from Matérn covariance.
